# Supplementary material for: The causal relationship between the human gut microbiota and pyogenic arthritis: a Mendelian randomization study
Source: Front Cell Infect Microbiol. 2024 Nov 26;14:1452480. doi: 10.3389/fcimb.2024.1452480 (PMC11629706; doi:10.3389/fcimb.2024.1452480)
Supplement: Supplementary file 1 [file DataSheet1.zip › mendelian test/ebi-a-GCST90027718.csv_forest.pdf]

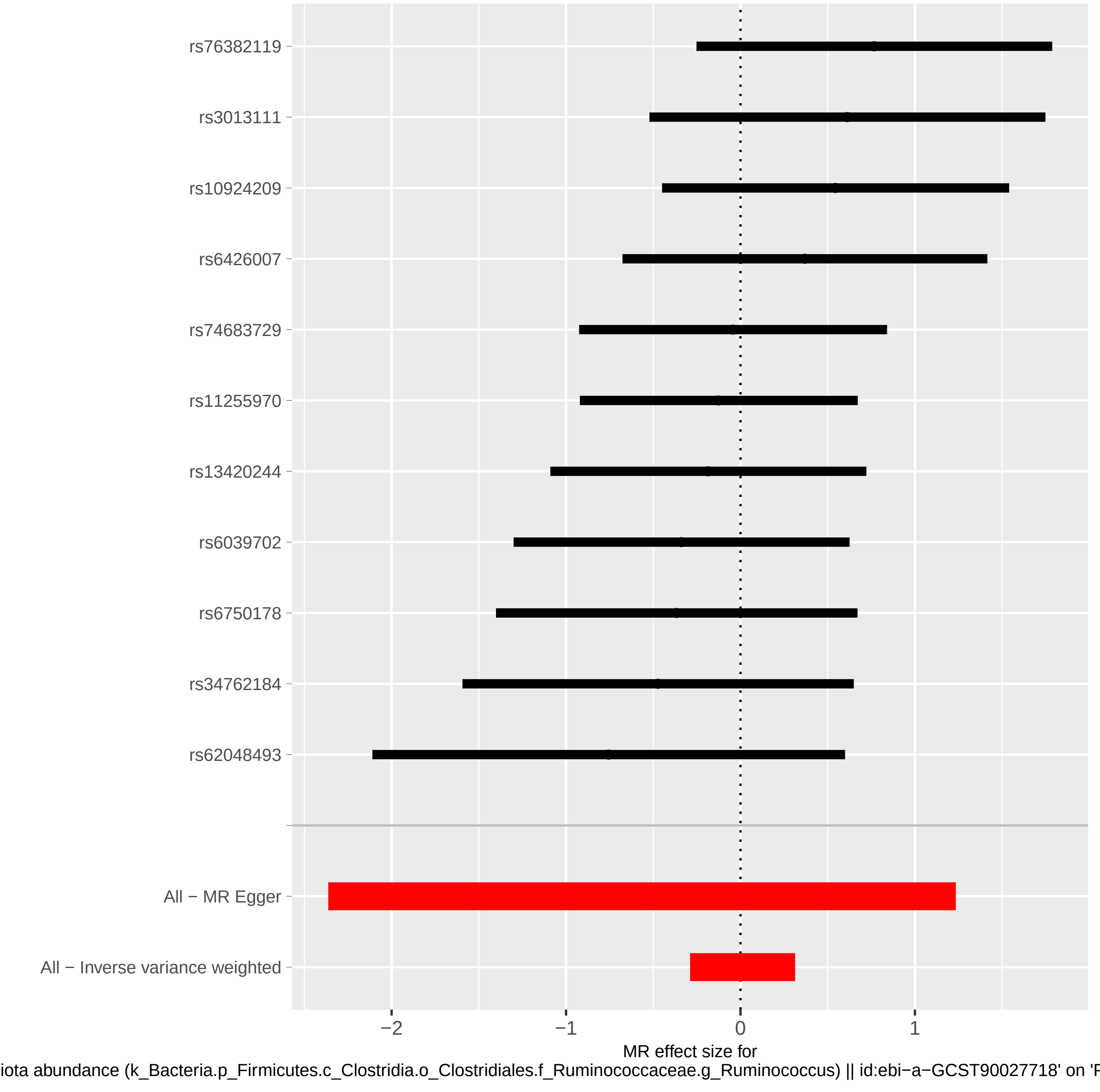
rs76382119
rs3013111
rs10924209
rs6426007
rs74683729
rs11255970
rs13420244
rs6039702
rs6750178
rs34762184
rs62048493
All − MR Egger
All − Inverse variance weighted
−2
−1
0
1
MR effect size for
iota abundance (k_Bacteria.p_Firmicutes.c_Clostridia.o_Clostridiales.f_Ruminococcaceae.g_Ruminococcus) || id:ebi−a−GCST90027718' on 'F
